# Analysis of Genome-Wide Monoallelic Expression Patterns in Three Major Cell Types of Mouse Visual Cortex Using Laser Capture Microdissection

**DOI:** 10.1371/journal.pone.0163663

**Published:** 2016-09-23

**Authors:** Chia-Yi Lin, Shih-Chuan Huang, Chun-Che Tung, Chih-Hsuan Chou, Susan Shur-Fen Gau, Hsien-Sung Huang

**Affiliations:** 1 Graduate Institute of Brain and Mind Sciences, College of Medicine, National Taiwan University, Taipei, Taiwan; 2 Department of Psychiatry, College of Medicine, National Taiwan University, Taipei, Taiwan; 3 Clinical Center for Neuroscience and Behavior, National Taiwan University Hospital, Taipei, Taiwan; 4 Neurobiology and Cognitive Science Center, National Taiwan University, Taipei, Taiwan; 5 Ph.D. Program in Translational Medicine, National Taiwan University and Academia Sinica, Taipei, Taiwan; 6 Neurodevelopment Club in Taiwan, Taipei, Taiwan; Florida State University, UNITED STATES

## Abstract

Genomic imprinting is an epigenetic mechanism causing monoallelic expression in a parent-of-origin-specific manner. Disruption of imprinted genes causes various neurological and psychiatric disorders. However, the role of imprinted genes in the brain is largely unknown. Different cell types within distinct brain regions can influence the genomic imprinting status, but imprinted genes in single cell types within distinct brain regions have not been characterized on a genome-wide scale. To address this critical question, we used a multi-stage approach, which combined genetically engineered mice with fluorescence-based laser capture microdissection (LCM) to capture excitatory neurons, inhibitory neurons and astrocytes as single cells in layer 2/3 of mouse visual cortex. RNA sequencing determined parental expression patterns on a genome-wide scale in the captured cells within specific brain regions. The expression level of cell-type-specific genes for excitatory neurons (13 genes), inhibitory neurons (16 genes) and astrocytes (20 genes) confirmed the LCM-captured cells maintained their cellular identities. The parent-of-origin-specific expression pattern of imprinted genes, including maternally expressed *Meg3* and paternally expressed *Peg3*, provided evidence that the status of known imprinted genes was also maintained. Although our platform remains to be improved, our findings demonstrate the parental expression pattern can be analysed not only at the level of a single cell type but also at the level of specific cortical layers. Our approach has the potential to reveal novel regulatory modules associated with plasticity through genomic imprinting mechanisms in different cell types, not only in the visual cortex but also in other brain regions.

## Introduction

The genomes inherited from each parent are not functionally equivalent due to genomic imprinting, which is established during gametogenesis and maintained throughout life [1–3]. Genomic imprinting is an epigenetic process through which monoallelic gene expression occurs in a parent-of-origin-specific manner. Genomic imprinting predominantly occurs in the brain [3]. Understanding genomic imprinting is limited not only because it is spatiotemporally dynamic, but also due to challenges associated with identifying environmental-, gender-, species-, cell-type- or gene isoform-specific parental expression effects [3–5]. Dysregulation of imprinted genes causes various neurological and psychiatric disorders such as autism spectrum disorder [6, 7], Angelman syndrome [8, 9], Prader-Willi syndrome [10], Rett syndrome [11], Turner’s syndrome [12], and myoclonus-dystonia syndrome [13]. Accordingly, there is an unmet need to identify imprinted genes in the brain and to understand their underlying physiological function. This is complicated by the heterogeneous nature of brain tissues and the multifaceted regulation of genomic imprinting. However, it is particularly critical to comprehensively determine parent-of-origin-specific gene expression in the brain, at least at a cellular resolution within a distinct brain region. Despite the importance of genomic imprinting in brain function, the numbers and identities of imprinted genes that have been proposed are still debated due to the heterogeneity of the brain and the complexity of screening techniques and statistical analysis [14–16].

To the best of our knowledge, an attempt to systematically identify imprinted genes at a cellular level within a distinct brain region has not been conducted due to the challenging and demanding techniques required. Many imprinted genes such as *Ube3a* [17–19], *Commd1* [5, 20], and *Snx14* [5] are imprinted in neurons but not in glia cells. It is also well established that different brain regions can influence the genomic imprinting status. For example, *Yipf6* shows a maternal bias in the preoptic area of the thalamus, and a paternal bias in the medial prefrontal cortex [15]; *Il18* shows a maternal bias in the medial prefrontal cortex, but no parental preference in the preoptic area of the thalamus [15]. These findings suggest many other genes could exhibit cell-type-specific imprinting within distinct brain regions.

To address this critical question, we employed a multi-stage approach focusing on the mouse visual cortex. The circuitry between the layers of mouse visual cortex has been well-characterized [21, 22]. Our methodology enabled us to identify a parent-of-origin-specific expression pattern on a genome-wide scale in the mouse visual cortex with cellular resolution. We used a strategy which coupled fluorescence-based laser capture microdissection (LCM) with RNA sequencing (RNA-Seq) to comprehensively profile the genomic imprinting status in the principal cell types of the mouse visual cortex on a genome-wide scale. The LCM-captured cells maintained their cellular identities and genomic imprinting status, which demonstrated the specificity and reliability of our approach. Although refinement of this multi-stage approach will improve the quality of the data, our findings provide the first evidence that a parental expression pattern in the mouse visual cortex can be analysed not only for an individual cell type, but also in a specific cortical layer of the brain. Our approach has the potential to uncover novel regulatory modules associated with plasticity in the visual cortex through genomic imprinting mechanisms in different cell types, which could also be applied to other brain regions.

## Materials and Methods

### Mice

**Fig 1:**
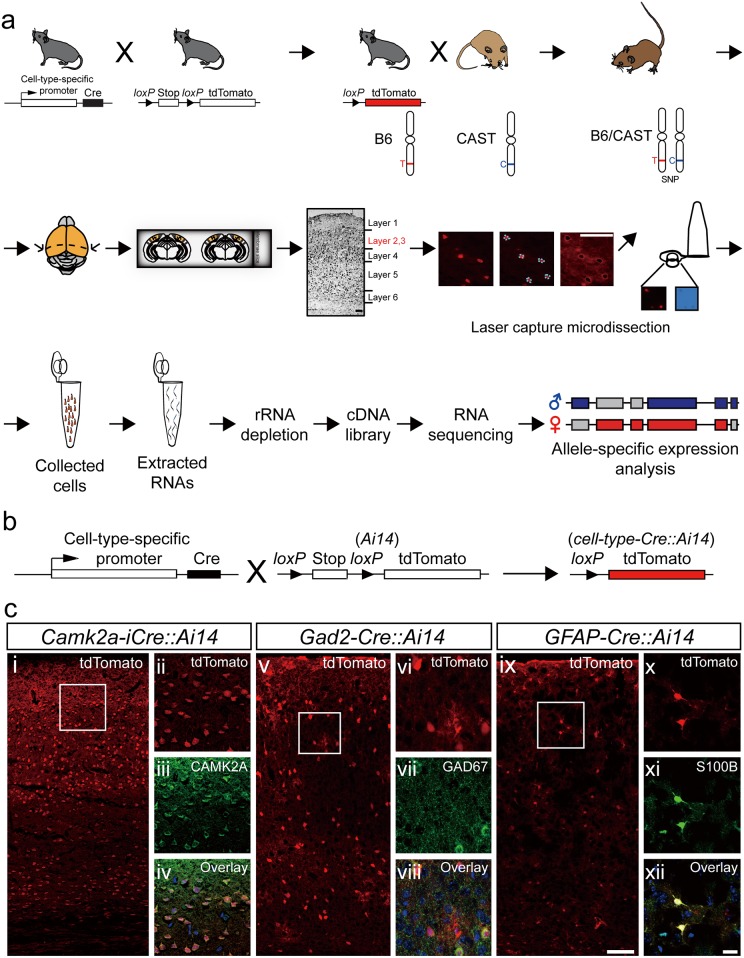
Process for detection and isolation of single cells from layer 2/3 of mouse visual cortex. (**a**) Schematic flowchart of the experimental design. **Top**: mating strategy to obtain B6/CAST mice. **Middle**: 7 μm cryostat sections from P28 mice were cut and single cells were identified for laser capture microdissection (LCM) as excitatory neurons, inhibitory neurons or astrocytes in layer 2/3 of the visual cortex. Scale bar, 50 μm. **Bottom**: LCM single cells were collected and RNA was extracted. A cDNA library, was generated for each cell type followed by RNA-Seq, and allele-specific expression in the cellular transcriptome was analyzed. (**b**) Schematic diagram showing cross of cell-type-specific Cre lines with a Cre/loxP reporter line (*Ai14*). (**c**) Confirmation of each specific cell type captured by LCM in Cre reporter cell lines. **Left panels *(i***, ***v***, ***ix)***: Representative images of the visual cortex from the three cell-type-specific Cre mouse lines. Cells are visualized with tdTomato (red). Cell-type specific Cre reporter mouse lines are indicated above each image. Squares indicate areas enlarged in left panel. Scale bar, 100 μm. **Right panels** (***ii-iv***, ***vi-viii***, ***x-xii)***: Enlarged images of cells labeled with antibodies (green) to anti-CAMK2A (excitatory neurons), anti-GAD2 (for inhibitory neurons) or anti-S100B (for astrocytes). Double-labeled cells (overlay) confirmed cell type of red fluorescent cells from the cell-type-specific Cre reporter lines. Sections were counterstained with DAPI (blue). Scale bar, 20 μm.

Mice were group-housed in ventilated cages, given access to food (PicoLab^®^ Rodent Diet 20, 5053) and water *ad libitum* and maintained on a 12-h light/dark cycle (lights off at 8 pm). All experimental procedures were approved by the National Taiwan University College of Medicine and College of Public Health Institutional Animal Care and Use Committee (IACUC) and were performed in strict accordance with National Institutes of Health guidelines for the care and use of laboratory animals. *Camk2a-iCre* mice (C57BL/6J background) were used for excitatory neurons; mice were generated in the laboratory of G. Schütz [23] and provided by the laboratory of C.-K. J. Shen. *Gad2-Cre* mice (stock number: 010802, C57BL/6J background) were used for inhibitory neurons and *Gfap-Cre* mice (stock number: 012886, C57BL/6J background) were used for astrocytes; both were purchased from the Jackson Laboratories, Bar Harbor, Maine. The full strain name of *Ai14* Cre reporter mice [24] is B6.Cg-*Gt(ROSA)26Sor*^*tm14(CAG-tdTomato)Hze*^/J (stock number: 007914), which were provided by the laboratory of S.L. Lin. C57BL/6J (B6) mice (stock number: 000664) and CAST/EiJ (CAST) mice (stock number: 000928) were purchased from Jackson Laboratories. To obtain cell-type-specific fluorescent cells, we bred cell-type-specific Cre-expression mice and Cre-reporter mice with a B6 background. Next, we crossed the mutant mouse (B6) with a wild-type mouse in CAST background [F1 initial cross (F1i); CAST wild-type mother x B6 mutant father] (Fig 1a, top). This breeding produced offspring with a B6 and CAST background and red fluorescent signals in specific cells. The B6/CAST mixed background has the advantage of yielding more identifiable single nucleotide polymorphisms (SNPs). To confirm our observations from F1i, we performed a reciprocal cross [F1 reciprocal cross (F1r): B6 mother x CAST father]. Offspring were euthanized at postnatal day 28 (P28) with 1% isoflurane followed by decapitation, and all efforts were made to minimize suffering. Brains were collected and processed according to the specific protocols described below.

### Genotyping

PCR was performed using *Camk2a-iCre*/F (5’-CTC TGA CAG ATG CCA GGA CA-3’), *Camk2a-iCre*/R (5’-TGA TTT CAG GGA TGG ACA CA-3’), *Gad2-Cre*-WT/F (5’-CTT CTT CCG CAT GGT CAT CT-3’), *Gad2-Cre*-Common (5’-CAC CCC ACT GGT TTT GAT TT-3’), *Gad2-Cre*-MT/F (5’-AAA GCA ATA GCA TCA CAA ATT TCA-3’), *Gfap-Cre*/F (5’-AAA ATT TGC CTG CAT TAC CG-3’), *Gfap-Cre*/R (5’-ATG TTT AGC TGG CCC AAA TG-3’), *Ai14 WT*/F (5’-AAG GGA GCT GCA GTG GAG TA-3’), *Ai14 WT*/R (5’-CCG AAA ATC TGT GGG AAG TC-3’), *Ai14 mutant*/F (5’-GGC ATT AAA GCA GCG TAT CC-3’), *Ai14 mutant*/R (5’-CTG TTC CTG TAC GGC ATG G-3’). The PCR cycling conditions were as follows: for *Camk2a-iCre* primers; initial denaturation at 94°C for 3 min followed by 35 cycles of 94°C for 30 s, 56°C for 30 s and 70°C for 60 s and a final extension at 72°C for 2 min; for *Gad2-Cre* primers; initial denaturation at 94°C for 3 min followed by 35 cycles of 94°C for 30 s, 55°C for 30 s and 72°C for 30 s and a final extension at 72°C for 2 min; for *Gfap-Cre* primers; initial denaturation at 94°C for 2 min followed by 35 cycles of 94°C for 30 s, 55°C for 30 s and 72°C for 20 s and a final extension at 72°C for 5 min; for *Ai14 WT and mutant* primers; initial denaturation at 94°C for 3 min followed by 35 cycles of 94°C for 20 s, 61°C for 30 s and 72°C for 30 s and a final extension at 72°C for 2 min. The size of PCR product is 381 bp for *Camk2a-iCre* primers, 250 bp for *Gad2/WT* primers, 352 bp for *Gad2/MT* primers, 350 bp for *Gfap-Cre* primers, 297 bp for *Ai14 WT* primers, 196 bp for *Ai14 MT* primers. Amplification was performed on a C1000 Touch Thermal Cycler (Bio-Rad).

### Immunofluorescence staining

For immunofluorescence staining, P28 brains were immersion-fixed with 4% paraformaldehyde in 0.1 M phosphate buffer (PB) (pH 7.4) for 12 h and then cryoprotected with 30% sucrose in 0.1 M PB at 4°C overnight. Fixed brains were embedded in O.C.T. compound (Surgipath, FSC 22). Cryostat sections (7 μm) were cut with a cryostat (Leica, CM3050 S) collected on glass slides, permeabilized with 0.3% Triton X-100 in 1X phosphate buffer saline (PBS) for 30 min at room temperature, and blocked in 1X PBS containing 5% goat serum and 0.3% Triton X-100 for 1 h. Sections were further incubated with anti-CAMK2A antibody (1:500, Acris Antibodies, AM 12015PU-S) at 4°C overnight or anti-GAD67 antibody (1:800, Millipore, MAB5406) at 4°C overnight, or anti-S100 beta antibody (1:500, Abcam, ab52642) at room temperature for 1 h, and then incubated with Alexa Fluor 488 goat anti-mouse IgG1 (γ1) antibody (1:500, Invitrogen, A21121 for CAMK2A) or Alexa Fluor 488 goat anti-mouse IgG2a antibody (1:500, Invitrogen, A21131 for GAD67) or Alexa Fluor 488 goat anti-rabbit IgG (H+L) antibody (1:500, Invitrogen, A11008 for GFAP) and DAPI (1:10,000, Invitrogen, D-1306) for 2 h at room temperature. Sections were washed in 1X PBS containing 0.1% Triton X-100 three times (5 min/wash) following incubations with primary and secondary antibodies. Sections were coverslipped with Fluoromount aqueous mounting medium (Sigma, F4680) and images were acquired using a Zeiss LSM 780 confocal microscope. Montage images were acquired with a 20X/0.8 NA objective and enlarged images were acquired with a 63X/1.4 NA objective.

### Fluorescence-based laser capture microdissection

P28 mouse brains were cut in half coronally and immersed in RNAlater stabilization solution (Ambion, AM7021) at 4°C for 1 h. A minimum of 6 h fixation is required to maintain a fluorescent signal during laser capture with 7 μm-thick cryostat sections of brain tissues. Therefore, brains were fixed in 4% paraformaldehyde (Sigma, SI-P6148) in 0.1 M PB at 4°C for 6 h followed by cryoprotection with 30% sucrose (J.T.-Baker, JT-4097-04) in 0.1 M PB at 4°C overnight. Brains were placed into a cryomold, embedded with O.C.T. compound (Surgipath, FSC 22) and frozen in a pre-chilled bath of isopentane (Sigma, AL-M32631) cooled with dry ice. To avoid obtaining more than one cell per capture, frozen brains were sectioned at a thickness of 7 μm with a cryostat (Leica, CM3050 S) and collected on membrane slides (Zeiss, 415190-7041-000). Sections were air-dried and dehydrated by performing one 30 second-dip in 50% ethanol (J.T. Baker, JT-8006-05) in DEPC (Sigma, D5758)-treated water, one 10 second-dip in 95% ethanol in DEPC-treated water, two 30 second-dips in 100% ethanol and two 2 minute-dips in 100% xylene (J.T. Baker, JT-9490-03). Red fluorescent positive cells in layer 2/3 of primary visual cortex were captured using a laser capture microdissection system (Zeiss, PALM MicroBeam) and collected into AdhesiveCap 500 opaque PCR tubes (Zeiss, 415190-9201-000). Layer 2/3 was defined by its relative location to all six layers of primary visual cortex [25, 26]. Tubes were replaced every hour. A total of 6000 cells per type were captured for further processing (Fig 1a, middle). In preliminary studies, we determined a minimum of 6000 cells per type was required for RNA-Seq analysis. A flowchart of the overall process of obtaining the LCM-captured cells is shown in Fig 1a. We also determined a minimum of 2000 cells per type was required for the confirmation of at least two imprinted genes with Sanger sequencing (S1 Fig).

### RNA isolation, library preparation and sequencing

Total RNA was extracted from the pooled LCM-captured cells with the NucleoSpin totalRNA FFPE XS kit (Macherey-Nagel, REF740969) with minor modifications. In short, cells in the cap were covered with lysis buffer, incubated at 56°C for 15 min and then spun down. Extracted RNA was bound onto the column with binding buffer and genomic DNA was digested for 15 min in rDNase incubation solution. After two washes, RNA was eluted with 10 μl of RNase-free water. rRNA was removed from the purified RNA with the Ribo-Zero Magnetic Gold Kit (Epicentre, MRZG126). The purified RNA was amplified with the SMARTer Universal Low Input RNA Kit for Sequencing (Clontech, 634938) and libraries were generated with the Low Input Library Prep Kit (Clontech, 634947). Libraries were sequenced on an Illumina HiSeq2000 (100 PE bp) at the Welgene Biotech Company, which generated 6 Gb of data per sample. RNA was quantified and qualitated with the Bioanalyzer 2100 (Agilent Technologies) using a RNA 6000 Pico LabChip (Agilent, 5065–4401). DNA was quantified and qualitated with an Agilent 2200 TapeStation system using High Sensitivity D1000 ScreenTape (Agilent, 5067–5584) and High Sensitivity D1000 Reagents (Agilent, 5067–5585).

### RNA-sequencing (RNA-Seq) analysis

To analyze the RNA-Seq data from the three cell types, qualified sequencing reads were obtained by filtering the generated sequences (S2 Fig and S1 Table). Per base sequence quality and per sequence quality scores from RNA-Seq data were analyzed by FastQC software (http://www.bioinformatics.babraham.ac.uk/projects/fastqc/). Subsequently, Trimmomatics was implemented to trim or remove the reads according to the quality score [27]. Qualified reads after filtering low-quality data were analyzed using TopHat/Cufflinks for gene expression estimation [28]. The gene expression level was calculated as FPKM (Fragments Per Kilobase of transcript per million mapped reads). The reference genome and gene annotations were retrieved from the Ensembl database. The Genome Analysis Toolkit (GATK) was used for SNP finding and genotype determination. MMSEQ was applied for Allelic-Specific-Expression (ASE) analysis. Monoallelic gene expression was defined as log(Ma/Pa) larger than 0.5 or less than -0.5. The identities of monoallelically-expressed genes in each cell type are shown in S2 Table.

### Quantitative real-time PCR (Q-RT-PCR)

Total RNA was extracted from LCM-captured excitatory neurons and astrocytes with the NucleoSpin miRNA kit (Macherey-Nagel, 740971). Q-RT-PCR was performed with a StepOnePlus Real Time PCR System (Applied Biosystem) using the SuperScript III Platinum SYBR Green One-Step qPCR Kit w/ROX (Invitrogen, 11746). The PCR cycling condition was as follows: initial denaturation at 95°C for 5 min followed by 50 cycles of 95°C for 15 s and 60°C for 30 s and a final extension at 40°C for 1 min. Ct values were generated by StepOne Software version 2.2.2. The expression level of each gene was normalized to *B2m*. All primer sequences are provided in S3 Table. The PCR product was purified with NucleoSpin Gel and PCR Clean-up kit (Macherey-Nagel, 740609) and analysed by Sanger sequencing.

## Results

### A multi-stage approach analysed parent-of-origin-specific monoallelic expression in individual cell types of the mouse visual cortex

This study employed a multi-stage approach to identify parent-of-origin-specific monoallelic expression in different cell types of the mouse visual cortex on a genome-wide scale. We targeted three major cell types: excitatory neurons, inhibitory neurons and astrocytes within distinct regions of the mouse visual cortex and resolved parent-of-origin-specific monoallelic expression patterns. To identify the individual cell types, we took advantage of the Cre-LoxP system. Cell-type-specific Cre mice were mated with td-Tomato Cre-reporter *Ai14* mice (Fig 1a, top). Single cells with red fluorescent signals from layers 2/3 of the visual cortex were isolated with laser capture microdissection (LCM) (Fig 1a, middle). We focused on these two layers because this is where plasticity of the visual cortex occurs [21, 22]. Next, to distinguish parent-of-origin-specific monoallelic expression, we took advantage of single nucleotide polymorphisms (SNPs), which were identified from the genomic DNA of C57BL/6J (B6) and CAST/EiJ (CAST) strain mice. Both have very distant sub-strains; therefore, it is much easier to distinguish the two sub-strain SNPs. Finally, RNAs were extracted from LCM-captured cells, which were then depleted of ribosomal ribonucleic acids (rRNAs). A cDNA library was constructed from the rRNA-depleted RNAs and further processed with genome-wide RNA-sequencing (RNA-Seq) followed by allele-specific expression analysis (Fig 1a, bottom).

### Cell-type-specific CRE expression is robust in the mouse visual cortex

Our multi-stage approach employed cell-type-specific Cre mice. We used *Camk2a-iCre* mice for excitatory neurons, *Gad2-Cre* mice for inhibitory neurons, and *GFAP-Cre* mice for astrocytes. To obtain red fluorescent signals in the cells, the cell-type-specific Cre mice were mated with Cre-reporter mice (*Ai14)*, offspring were bred with CAST/EiJ mice, and the resulting offspring were used for LCM (Fig 1a and 1b). Red fluorescent cells in the visual cortex confirmed the presence of the three distinct cell types (Fig 1ci, 1cv and 1cix). Each cell type was further validated by immunofluorescence with cell-type-specific antibodies: α-CAMK2A for excitatory neurons; α-GAD2 for inhibitory neurons; and α-S100B for astrocytes (Fig 1ciii, 1cvii and 1cxi). Double-labeled cells confirmed gene and protein expression in *Camk2a-iCre*::*Ai14*, *Gad2-Cre*::*Ai14* and *Gfap-Cre*::*Ai14* mice (Fig 1civ, 1cviii and 1cxii). Our results demonstrate that cell-type-specific expression can be detected in the mouse visual cortex using this Cre-LoxP system, and the level of expression is robust.

### LCM-captured cells maintain their cellular identities in distinct layers of visual cortex

**Fig 2:**
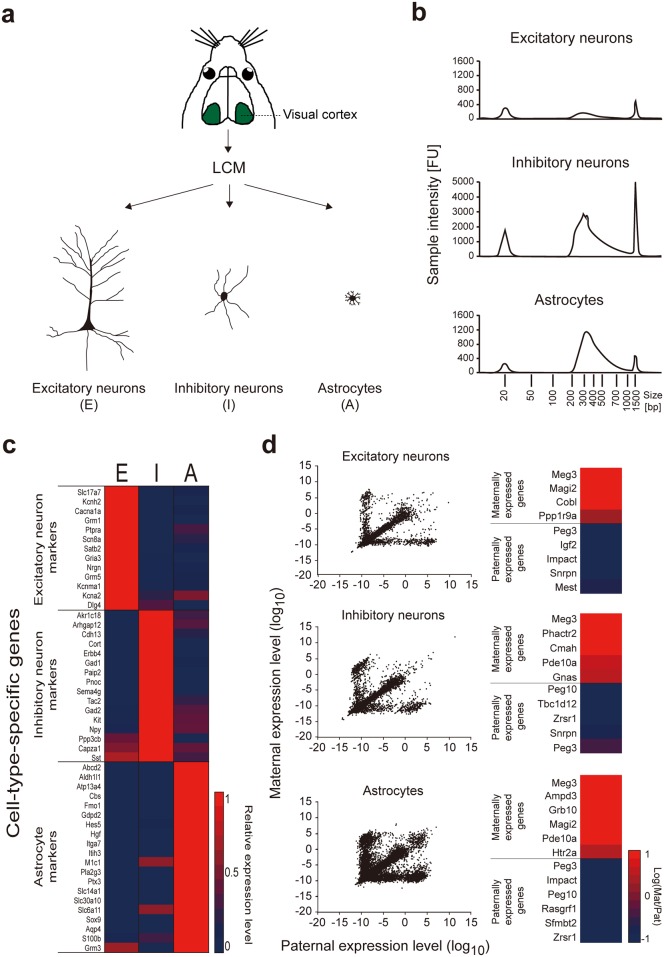
Cellular identity and genomic imprinting status were maintained in LCM-captured cells of mouse visual cortex. (**a**) Schematic diagram of the mouse visual cortex and the three cell types captured with LCM: excitatory neurons (E), inhibitory neurons (I) and astrocytes (A). (**b**) Quality of the cDNA library was evaluated by the fragment peak size and concentration of cDNA using the TS HS D1000 (Agilent) assay. Top: excitatory neurons; fragment peak size = 284 bp; concentration = 7 nM. Middle: inhibitory neurons; fragment peak size = 293 bp; concentration = 180 nM. Bottom: astrocytes; fragment peak size = 327 bp; concentration = 42 nM. A minimum fragment peak size of 240 bp and concentration of 4 nM was required for performing RNA-Seq. (**c**) Heat map showing expression of cell-type-specific genes, which confirmed the identities of the LCM captured cells were excitatory neurons, inhibitory neurons, and astrocytes. (**d**) **Left panel**: Excitatory neurons (top), inhibitory neurons (middle) and astrocytes (bottom) exhibited similar patterns of monoallelic gene expression. **Right panel:** Known maternal and paternal imprinted genes in the three cell types. The imprinting status of known imprinted genes was maintained in all cell types. Excitatory neurons were obtained from offspring of a female CAST x male B6 cross. Inhibitory neurons and astrocytes were obtained from offspring of a female B6 x male CAST cross.

We focused on three major cell types in the mouse visual cortex for implementing fluorescence-based LCM: excitatory neurons (E), inhibitory neurons (I), and astrocytes (A) (Fig 2a). We extracted RNAs from the LCM-captured cells and then depleted rRNAs. We converted the rRNA-depleted RNAs into a cDNA library and the resulting concentration and cDNA fragment size (Fig 2b) met the criteria for further analysis with RNA-Seq. RNA-Seq analysis demonstrated the LCM-captured cells exhibited high expression levels of cell-type-specific genes for excitatory neurons, inhibitory neurons, and astrocytes (Fig 2c). The appropriate genetic properties displayed by each cell type confirmed our methodology of obtaining cells from the visual cortex by LCM did not disrupt their cellular identities.

### LCM-captured cells maintain their genomic imprinting status associated with distinct layers of the visual cortex

To determine whether LCM-captured cells exhibited a parent-of-origin-specific expression pattern, we profiled monoallelic expression in the LCM captured cells. The three major cell types of the mouse visual cortex exhibited similar patterns of monoallelic gene expression: one diagonal line, which indicated equal expression of genes from paternal and maternal alleles, one cohort above the diagonal line, representing expression of genes from maternal alleles, and one cohort below the diagonal line, representing expression of genes from paternal alleles, which was seen in initial and reciprocal crosses (Fig 2d, left and S3 Fig). Unfortunately, the low density of genes in the inhibitory neurons and astrocytes of the initial crosses prevented any further analysis of initial crosses.

To further investigate whether monoallelically expressed genes are predominantly expressed in three major cell types of mouse visual cortex and whether there is any overlappability of monoallelically expressed genes between the three cell types in mouse visual cortex, we calculated the percent of monoallelically expressed genes in the three cell types and the overlappability of monoallelically expressed genes among them. Our results showed that, although the percentages of monoallelically expressed genes differed between excitatory neurons, inhibitory neurons and astrocytes, each cell type had a similar percentage from paternal and maternal alleles (S4 Table). The percent of monoallelically expressed genes was less than 12% regardless of allele source. Examination of the overlappability of these monoallelically expressed genes in these three cell types indicated the percent of overlap was less than 20% regardless of allele source (S4 Fig and S5 Table). Monoallelically expressed genes from S4 Fig and overlapped genes from S5 Table for each cell type are shown in S2 Table. Finally, known imprinted genes including maternally expressed *Meg3* and paternally expressed *Peg3* were detected in our LCM-captured cells regardless of cell type (Fig 2d, right), which indicated LCM-capturing did not alter the genomic imprinting status. We also confirmed the presence of a known imprinted gene, *Meg3* and a known neuron-specific imprinted gene, *Ube3a*, in our LCM-captured cells with Sanger Sequencing (S1 Fig), suggesting that RNA obtained from our method of LCM-capture can be validated by a second approach. These data suggest that monoallelic gene expression is prevalent in the brain transcriptome regardless of cell type.

## Discussion

Dysfunction of imprinted genes causes various neurological and psychiatric disorders but the role of genomic imprinting in the brain remains largely un-explored. Genomic imprinting status can be influenced by factors such as cell types, brain regions and developmental timing, resulting in inconsistency in the identities and numbers of imprinted genes in the brain [15, 16, 29]. Understanding brain function involves advanced knowledge of how the genome specifies diverse cell types [30, 31]. Here, we established a multi-stage approach to explore genomic imprinting status of three major cell types in mouse visual cortex: excitatory neurons, inhibitory neurons and astrocytes. Because plasticity occurs in layers 2/3, we focused on this brain region for our studies. LCM-captured cells exhibited the correct cellular identities, based on the expression of appropriate cell-type-specific genes, and maintenance of the genomic imprinting status associated with the brain region, which was confirmed by the status of known imprinted genes including maternally expressed *Meg3* and paternally expressed *Peg3*. Our studies provide the first evidence that parent-of-origin-specific monoallelic expression can be analysed at the level of an individual cell type as well as at the level of a specific cortical layer of a brain region on a genome-wide scale. These findings suggest this multi-stage approach can be used to understand parental expression patterns and the genomic imprinting status of individual cell types.

High-throughput sequencing has been an effective strategy for decoding neural transcriptomes and epigenomes on a genome-wide scale [32]. However, the brain is heterogeneous and cell types and brain regions can affect genomic imprinting status [15]. Therefore, there is an un-met need to perform RNA-Seq on individual cell types from distinct regions of the brain. Moreover, cell-type transcriptomics using next-generation transcript sequencing (RNA-Seq) is emerging as a very powerful tool for profiling cell-to-cell variability on a genomic scale. Application of RNA-Seq has changed our conceptual perception of diverse biological processes with broad implications for both clinical and basic research [33].

We developed a multi-stage approach for cell analysis by combining the use of genetically engineered mice with fluorescence-based LCM and subsequent RNA-Seq analysis. Laser capture microdissection is the only approach which affords isolation of a homogeneous cellular population from specific microscopic regions of heterogeneous tissue sections of the brain under direct microscopic visualization for analysis at the RNA level. RNA amplification and next-generation deep sequencing techniques enabled the use of a pool of total RNA from a few thousand cells to obtain a relatively thorough inventory of the transcriptome and imprintome. Characterizing the transcriptome and genomic imprinting status of three individual cell types from heterogeneous brain tissue demonstrated the feasibility of coupling fluorescent LCM with RNA-Seq.

The strength of our approach is monoallelic expression in an individual cell type could be determined within specific layers of the visual cortex. This resolution cannot be obtained with other methods such as fluorescence-activated cell sorting, which collects a population of fluorescent cells, or single-cell RNA-Seq combined with the Fluidigm C1 system [34]. Moreover, using the Cre-loxP system allows for collection of individual cell types without using an additional step, such as immunofluorescence staining to define cell types. The drawbacks of immunofluorescence staining are long incubation times and limited choices of good-quality cell-type-specific antibodies. Although our method is a significant step in allele-specific expression analysis of cell types, advancements in methodology will be required to improve the quality of the data. RNA-Seq analysis was limited by the number and quality of the cells obtained, which was the primary reason that only 1% (or less) of RNA-seq reads could be mapped. Capturing an adequate number of fixed cells (n = 6,000) for each cell type was time-consuming and labour-intensive. Second, detection of fluorescent proteins in 7-**μ**m thin sections demanded the use of 4% paraformaldehyde-fixed brains. Although thin sections decreased the possibility of collecting more than one cell from each capture, the RNA collected was comparatively low in quality (fragmented RNAs due to fixation) and quantity (~1ng/6,000 cells), which resulted in the low genome mappability of read sequences and limited further analysis (S1 Table). However, the purpose of our study was to show the strengths and limitations of this novel technique. Future improvements in proficiency should increase mappability.

This proof-of-principle study demonstrated our approach has the potential to determine parental expression patterns and investigate genomic imprinting status on a genome-wide scale in individual cell types isolated from distinct layers of the visual cortex. To the best of our knowledge, this is the first study demonstrating the feasibility of employing this technique as a means to identify monoallelic gene expression in individual cell types with high-resolution. Cell-type-specific imprinted genes have not been profiled on a genome-wide scale in the brain. The neuron-specific imprinted gene, *Ube3a*, was imprinted in our LCM-captured excitatory neurons (S1 Fig), which validated our approach. Our studies suggest that this procedure could be used not only for precise and meaningful analysis of transcriptional and genomic imprinting of cell types in other brain regions, but could also be used as an approach to evaluate differences between types of cells, between cells of healthy and unhealthy persons, or to measure changes in cells following therapeutic treatments.

Our methodology is in its infancy, and further analysis of the monoallelically-expressed genes observed in this study will be required. The genes identified could be parent-of-origin-specific, strain-specific (B6 or CAST) or random. Therefore, it is crucial that RNA-Seq be performed on the offspring from initial (CAST wild-type mother x B6 mutant father) and reciprocal (B6 mother x CAST father) mating. More importantly, further confirmation with a second approach such as Sanger sequencing, pyrosequencing and MassARRAY analysis is necessary. Expression quantitative trait loci (eQTLs) have been shown to be associated with gene regulation events of their target genes [35]. Differential DNA methylation defines genomic imprinting and allele-specific expression is the consequence of genomic imprinting. However, the expression from a single allele can result from two entirely independent processes through either cis-/trans-acting genetic variants or differential DNA methylation. Therefore, it would be of interest to evaluate these processes in LCM captured single cell types to determine causal consequences of monoallelic gene expression.

Parent-of-origin-specific allelic expression has previously been analyzed in the mouse brain although the findings have been controversial in the field of genomic imprinting [15, 16, 29]. Subsequent rigorous and thorough studies by Bonthuis et. al. [36] and Perez et. al. [37] demonstrate the complexity of genomic imprinting [36, 37]. The authors determined that there are different magnitudes of parentally biased gene expression, which can be influenced by age, organ, as well as brain region and cell type. Their work showed the significance of validating novel imprinted genes identified by RNA-Seq analysis with a second approach. Their findings emphasize the importance of specifying the conditions of the samples acquired when discussing the genomic imprinting status. Finally, their approach demonstrates the urgency to develop reliable methods to analyze parental expression patterns in single cell types of the brain, which was the purpose of the current study.

It has been shown that between 0.5% and 15% of autosomal genes exhibit random and dynamic monoallelic gene expression in a cell-type specific and developmental stage-dependent manner [38, 39]. Moreover, the monoallelic expression has considerable variation even among closely related cells [39]. This large variability of monoallelically expressed genes could partially explain the different percentage of monoallelically expressed genes in three major cells types of mouse visual cortex (S4 Table) and less overlappability of monoallelically expressed genes among three major cell types (S4 Fig, S2 and S5 Tables). It will be interesting to further examine monoallelic gene expression patterns across multiple cell types and different developmental stages in the brain. Through such examinations, we may know whether there is less variability of monoallelic gene expression in a cluster of cell types or at distinct developmental stages in the brain.

In summary, our results provide a genome-wide resolution of monoallelic expression in different cell types of layer 2/3 from mouse visual cortex and provide evidence for the feasibility of studying the genomic imprinting status in different cell types within distinct brain regions. Our methodology offers a significant advance in techniques for studying genomic imprinting in the brain, which could be applied for further study of functions of genomic imprinting within individual cells. The ability to investigate the transcriptome and imprintome in a single cell type could be utilized to provide therapeutic strategies for genomic imprinting-related brain disorders.

## Supporting Information

S1 Fig The neuron-specific imprinted gene, *Ube3a*, was validated in LCM-captured cells. (**a**), Schematic diagram for capturing excitatory neurons and astrocytes in the mouse visual cortex with LCM and further Sanger sequencing analysis. (**b**), Sanger sequencing was performed for *Meg3* in the LCM-captured excitatory neurons. (**c**), Sanger sequencing was performed for *Ube3a* in the LCM-captured excitatory neurons and astrocytes.
(TIF)

S2 Fig Quality control data from RNA-Seq experiments. Per base sequence quality (**a**) and per sequence quality scores (**b**) from six RNA-Seq data were analyzed by FastQC software. The background of the graph in the panel a divides the y axis into very good quality calls (green), calls of reasonable quality (orange), and calls of poor quality (red). An error is raised in the panel b if the most frequently observed mean quality is below 20. The initial cross was obtained by breeding a female CAST and male B6 mouse. The reciprocal cross was obtained by breeding a female B6 x male CAST mouse.
(TIF)

S3 Fig Allele-specific expression patterns in LCM-captured cells of mouse visual cortex. Allele-specific expression was analyzed in LCM-captured cells of mouse visual cortex. Excitatory neurons were obtained from offspring of a female B6 x male CAST cross. Inhibitory neurons and astrocytes were obtained from offspring of a female CAST x male B6 cross.
(TIF)

S4 Fig Overlappability of monoallelically expressed genes from three cell types of mouse visual cortex. Venn diagram analysis shows the overlappability of monoallelically expressed genes from maternal or paternal alleles (**a**), maternal alleles only (**b**), and paternal alleles only (**c**) in excitatory neurons (E), inhibitory neurons (I), and astrocytes (A) of mouse visual cortex. Excitatory neurons were obtained from offspring of a female CAST x male B6 cross. Inhibitory neurons and astrocytes were obtained from offspring of a female B6 x male CAST cross.
(TIF)

S1 Table Quantitation of mappability and total reads. (XLSX)

S2 Table Monoallelically expressed genes from three major cell types of mouse visual cortex. (XLSX)

S3 Table Primer sequence for Q-RT-PCR. (XLSX)

S4 Table Summary of proportion of monoallelically expressed genes in three major cell types of mouse visual cortex. (XLSX)

S5 Table Overlappability of monoallelically expressed genes from three major cell types of mouse visual cortex. (XLSX)
