# Supplementary figures and images for: Analysis of Genome-Wide Monoallelic Expression Patterns in Three Major Cell Types of Mouse Visual Cortex Using Laser Capture Microdissection
Source: PLoS One. 2016 Sep 23;11(9):e0163663. doi: 10.1371/journal.pone.0163663 (PMC5035046; doi:10.1371/journal.pone.0163663)

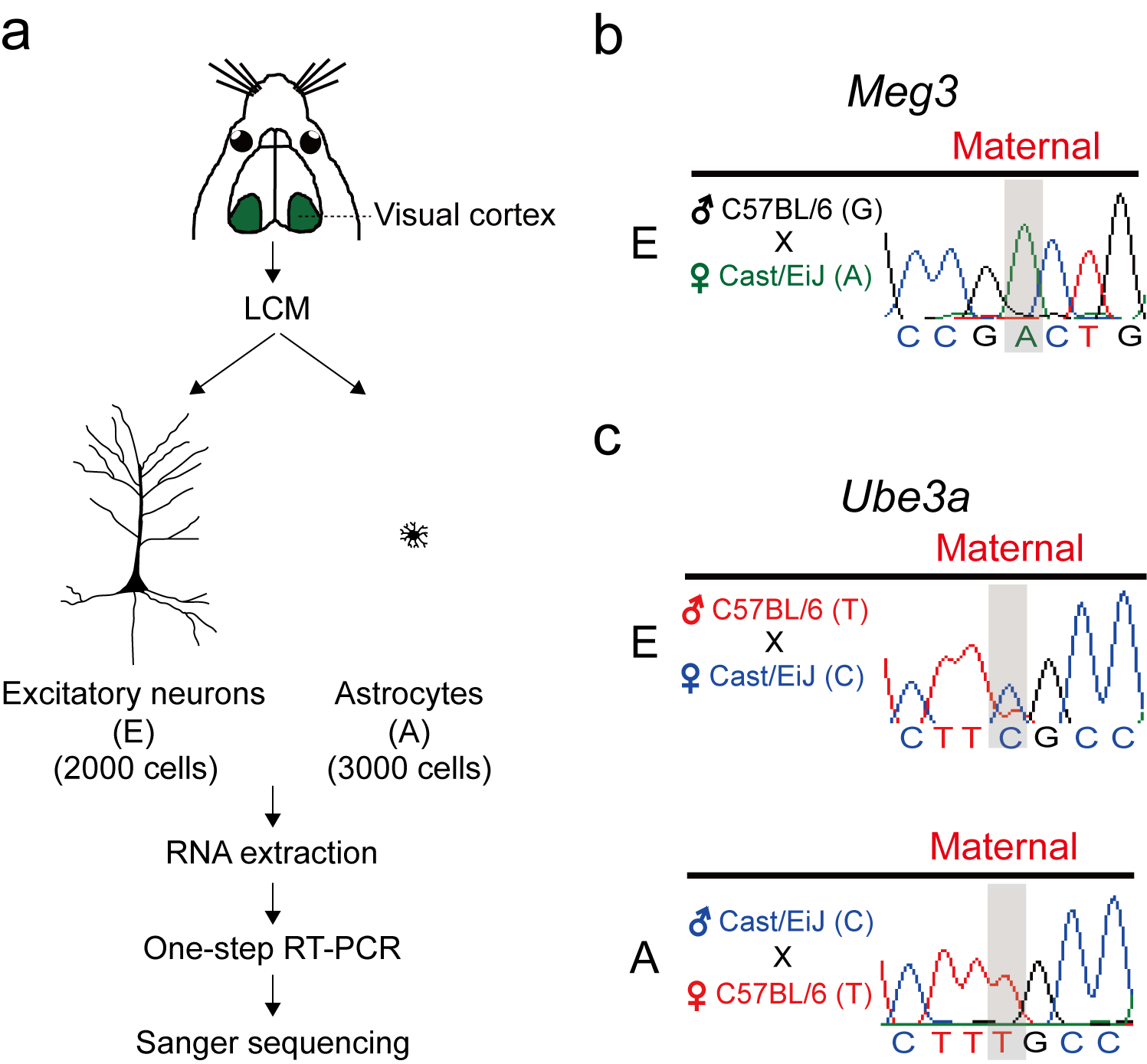

Supplement: S1 Fig — (a), Schematic diagram for capturing excitatory neurons and astrocytes in the mouse visual cortex with LCM and further Sanger sequencing analysis. (b), Sanger sequencing was performed for Meg3 in the LCM-captured excitatory neurons. (c), Sanger sequencing was performed for Ube3a in the LCM-captured excitatory neurons and astrocytes. (TIF) [file pone.0163663.s001.tif]

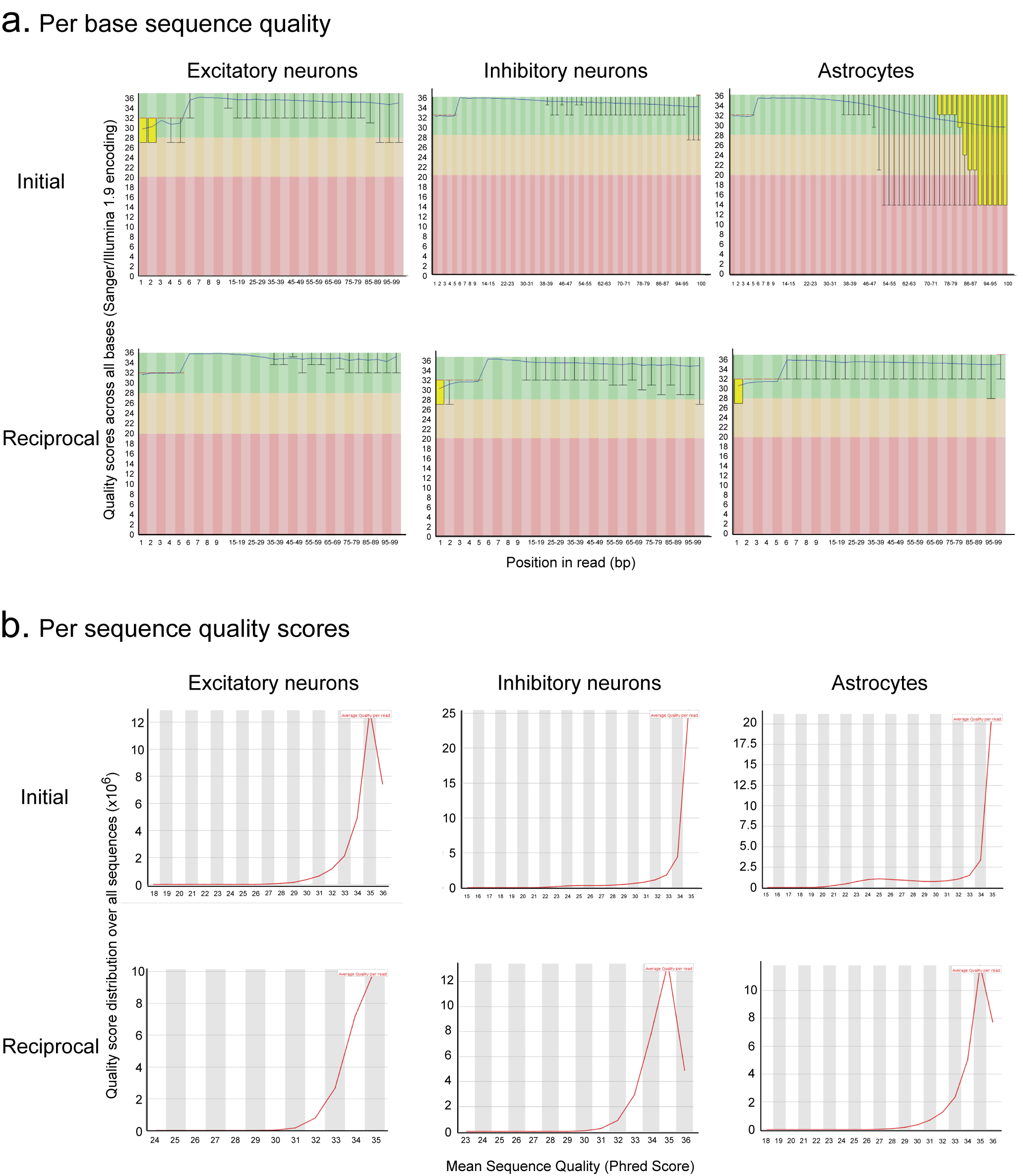

Supplement: S2 Fig — Per base sequence quality (a) and per sequence quality scores (b) from six RNA-Seq data were analyzed by FastQC software. The background of the graph in the panel a divides the y axis into very good quality calls (green), calls of reasonable quality (orange), and calls of poor quality (red). An error is raised in the panel b if the most frequently observed mean quality is below 20. The initial cross was obtained by breeding a female CAST and male B6 mouse. The reciprocal cross was obtained by breeding a female B6 x male CAST mouse. (TIF) [file pone.0163663.s002.tif]

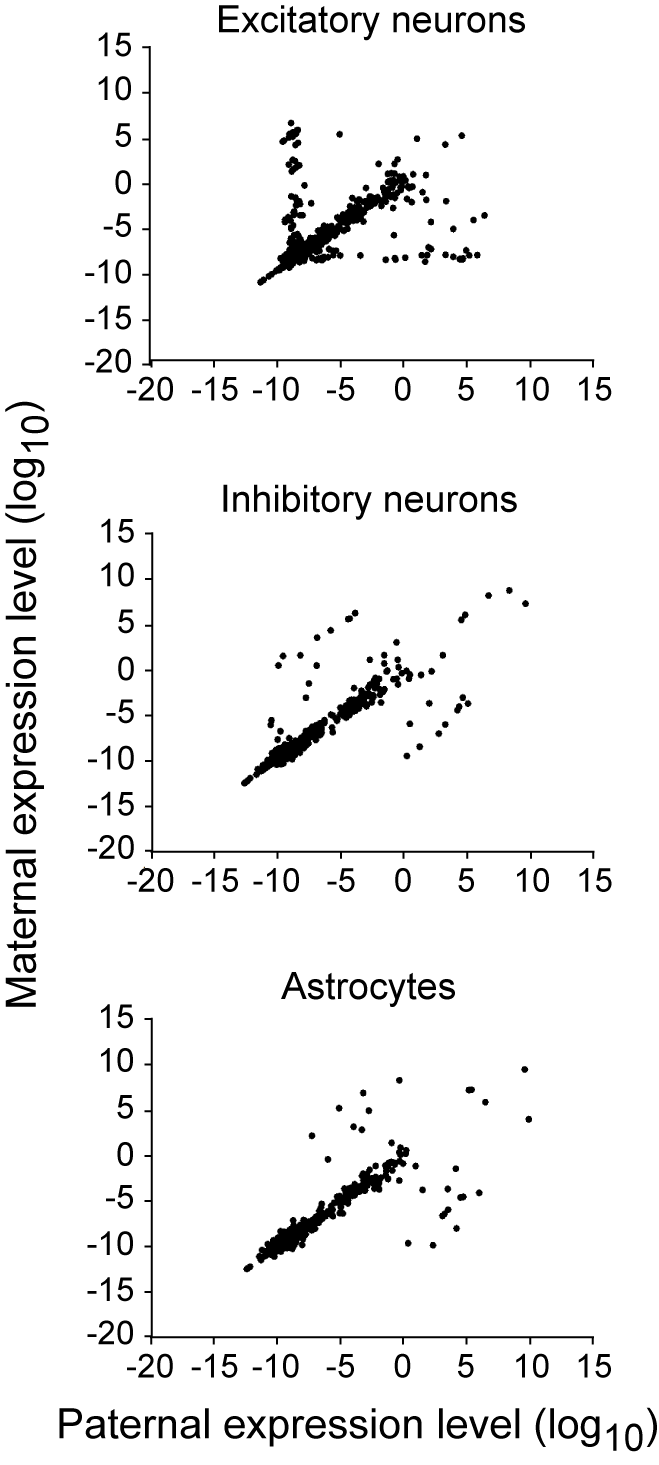

Supplement: S3 Fig — Allele-specific expression was analyzed in LCM-captured cells of mouse visual cortex. Excitatory neurons were obtained from offspring of a female B6 x male CAST cross. Inhibitory neurons and astrocytes were obtained from offspring of a female CAST x male B6 cross. (TIF) [file pone.0163663.s003.tif]

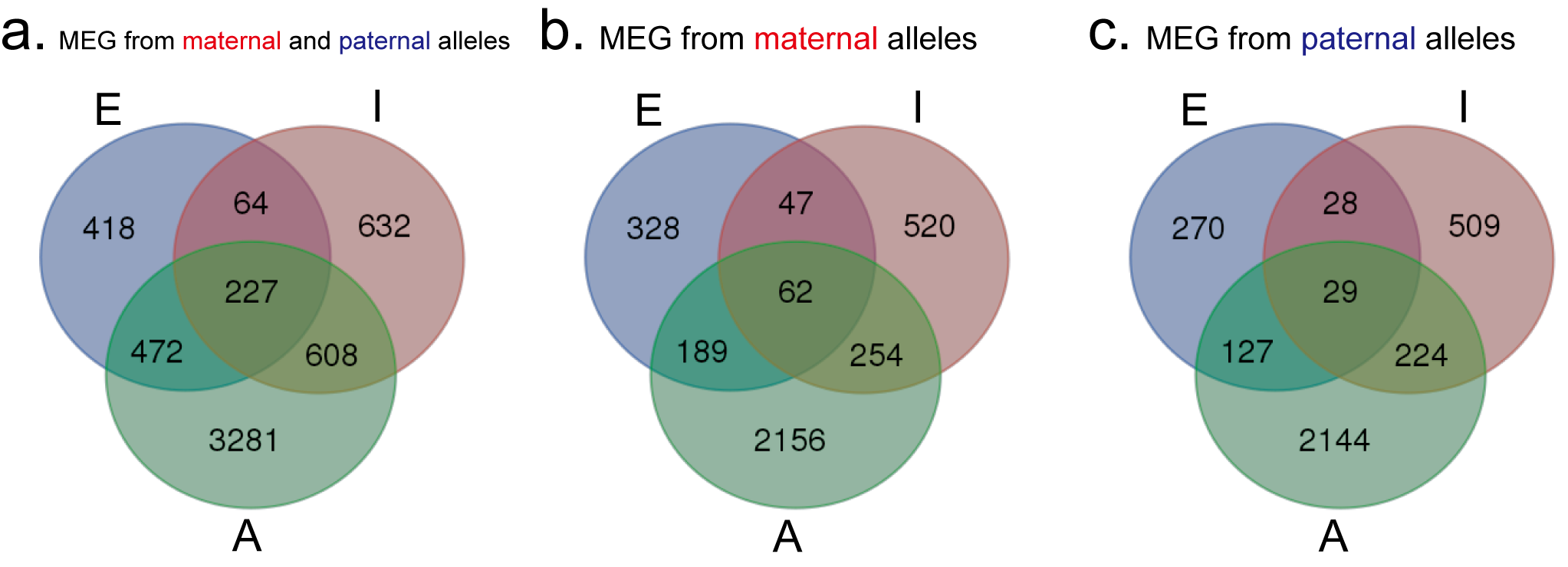

Supplement: S4 Fig — Venn diagram analysis shows the overlappability of monoallelically expressed genes from maternal or paternal alleles (a), maternal alleles only (b), and paternal alleles only (c) in excitatory neurons (E), inhibitory neurons (I), and astrocytes (A) of mouse visual cortex. Excitatory neurons were obtained from offspring of a female CAST x male B6 cross. Inhibitory neurons and astrocytes were obtained from offspring of a female B6 x male CAST cross. (TIF) [file pone.0163663.s004.tif]
